# Modifiable lifestyle, mental health status and diabetic retinopathy in U.S. adults aged 18–64 years with diabetes: a population-based cross-sectional study from NHANES 1999–2018

**DOI:** 10.1186/s12889-023-17512-8

**Published:** 2024-01-02

**Authors:** Bo Li, Chuandi Zhou, Chufeng Gu, Xiaoyun Cheng, Yujie Wang, Chenxin Li, Mingming Ma, Ying Fan, Xun Xu, Haibing Chen, Zhi Zheng

**Affiliations:** 1 Department of Ophthalmology, Shanghai General Hospital, Shanghai Jiao Tong University School of Medicine, National Clinical Research Center for Eye Diseases, Shanghai Clinical Research Center for Eye Diseases, Shanghai Key Clinical Specialty, Shanghai Key Laboratory of Ocular Fundus Diseases, Shanghai Engineering Center for Visual Science and Photomedicine, Shanghai Engineering Center for Precise Diagnosis and Treatment of Eye Diseases, 100 Haining Road, Hongkou District, Shanghai, 200080 China; 2 Department of Endocrinology and Metabolism, Shanghai 10th People’s Hospital, Tongji University, 301 Middle Yanchang Road, Jingan District, Shanghai, 200072 China

**Keywords:** Diabetic retinopathy, Lifestyles, Diabetes, NHANES

## Abstract

**Background:**

The relationship between integrated lifestyles, mental status and their impact on overall well-being has attracted considerable attention. This study aimed to evaluate the association between lifestyle factors, depression and diabetic retinopathy (DR) in adults aged 18–64 years.

**Methods:**

A cohort of 3482 participants diagnosed with diabetes was drawn from the National Health and Nutrition Examination Survey (NHANES) spanning the years 1999–2018. DR was defined based on self-reported diabetic retinopathy diagnoses by professional physicians, relying on Diabetes Interview Questionnaires. Subgroup analysis was employed to assess lifestyle and psychological factors between participants with DR and those without, both overall and stratified by diabetic duration. Continuous variables were analyzed using the student’s *t* test, while weighted Rao-Scott χ^2^ test were employed for categorical variables to compare characteristics among the groups.

**Results:**

Of the 3482 participants, 767 were diagnosed with diabetic retinopathy, yielding a weighted DR prevalence of 20.8%. Patients with DR exhibited a higher prevalence of heavy drinking, depression, sleep deprivation, and insufficient physical activity compared to those without DR. Furthermore, multivariable logistic regression analysis revealed that sleeping less than 5 h (OR = 3.18, 95%CI: 2.04–4.95, *p* < 0.001) and depression (OR = 1.35, 95%CI:1.06–1.64, *p* = 0.025) were associated with a higher risk of DR, while moderate drinking (OR = 0.49, 95%CI: 0.32–0.75, *p* = 0.001) and greater physical activity (OR = 0.64, 95%CI: 0.35–0.92, *p* = 0.044) were identified as protective factors.

**Conclusions:**

Adults aged 18–64 years with DR exhibited a higher prevalence of lifestyle-related risk factors and poorer mental health. These findings underscore the need for concerted efforts to promote healthy lifestyles and positive emotional well-being in this population.

**Supplementary Information:**

The online version contains supplementary material available at 10.1186/s12889-023-17512-8.

## Background

Diabetes has rapidly become one of the most prevalent diseases globally. While diabetes prevention is crucial, mitigating diabetic vascular complications that contribute significantly to considerable morbidity and mortality is an even more pressing concern [1]. Diabetic retinopathy (DR), a major microvascular complication, has now become a leading cause of blindness among working-age adults [2]. According to The Vision Loss Expert Group, DR ranks sixth among the most prevalent preventable causes of vision impairment, potentially impacting as many as 160.5 million people worldwide [3]. Consequently, the prevention and timely treatment of DR carry immense clinical significance.

Numerous publications have established the importance of not only controlling traditional metabolic factors such as hyperglycemia, hypertension and hyperlipemia, but also maintaining a healthy lifestyle as a crucial strategy for preventing DR [4–7]. Despite significant advances in the development of medications targeting traditional metabolic factors effectively preventing the occurrence of diabetic complications, the promotion lifestyle risk management remains an area where progress is needed, particularly among adults aged 18–64 years [8]. The prevalence of diabetes in this age group is anticipated to increase by 18% over the next decade, reaching 417 million by 2030 [9]. In addition to the substantial medical costs associated with diabetes, it may also result in work disability [10], with indirect economic losses potentially surpassing the expenses incurred for treatment [11]. Therefore, assessing diabetes-related lifestyle factors among adults aged 18–64 years is crucial for preventing DR. However, to our knowledge, research in this field is still lacking.

This study aims to assess the differences in lifestyle-related and mental factors among adults aged 16–64 years diagnosed with diabetes, both with and without DR, and to explore their association with the development of DR.

## Methods

### Data resource and study population

National Health and Nutrition Examination Survey (NHANES), an ongoing, biennial cross-sectional survey, utilized a complex multistage probability sampling design to investigate the nutritional and health status of the civilian, non-institutionalized U.S. population [12, 13]. Data collected involved physical examinations, laboratory tests, and questionnaires. Informed written consent was obtained from all participants, and the study received ethical approval from the National Center for Health Statistics. The research adhered to the Tenets of the Declaration of Helsinki.

**Fig. 1:**
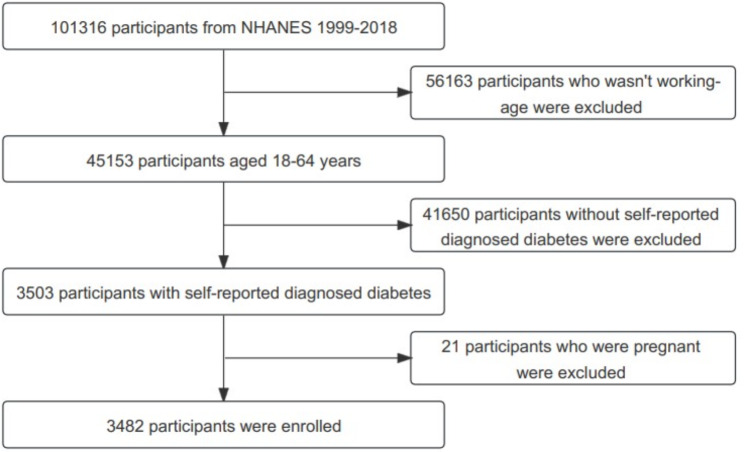
A comprehensive flow chart of the study design A total of 101,316 participants were enrolled in NHANES 1999–2018. 56163 participants who wasn’t working-age adults were excluded. 41650 participants without self-reported diabetes were excluded. After excluding 21 pregnant participants, 3482 participants were included.

Our study encompassed 3482 participants aged 18–64 years from NHANES 1999–2018, as illustrated in Fig. 1. All participants were non-pregnant and had received a diagnosis of diabetes from a physician or other health professional at 18 years of age or older (excluding diagnosis made solely during pregnancy)

### Definitions of diabetes, diabetic retinopathy and lifestyle-related factors

Diabetes in this study was defined based on self-reported diagnoses confirmed by professional physicians, rather than relying on glycosylated hemoglobin A1c (HbA1c) and self-reported results. This approach was selected due to the significant impact of diabetic duration as a risk factor for DR, with newly-diagnosed diabetes not adequately reflecting its substantial influence. Diabetic retinopathy was identified through self-reported diagnosed retinopathy, as confirmed by a dichotomous item as presented consistent with previous literature [14–17]. In the Diabetes Interview Questionnaires, participants diagnosed with diabetes were asked “Has a doctor ever told you/SP that diabetes has affected your/his/her eyes or that you/s/he had retinopathy (ret-in-op-ath-ee)?” Affirmative responses were considered indicative of self-reported DR. The duration of diagnosed diabetes was calculated as the age at examination minus the age at diagnosis, then subcategorized into three groups: 0–10 years, 11–19 years and ≥ 20 years.

Lifestyle-related variables in NHANES encompassed smoking status, drinking status, physical activity, healthy eating index (HEI), sleeping duration, and self-reported sleeping disorder. Smoking status (never smoker, former smoker, current smoker) was determined by using Smoking-Cigarette Use questionnaires, with never smoking defined as lifetime consumption of fewer than 100 cigarettes. Drinking status (no drinking, mild to moderate drinking, heavy drinking) was based on self-reported drinking frequency and quantity, following officially provided units (1 drink contains 14 g of ethanol) [18]. Mild to moderate drinking was defined as < 1 drink/day for women and < 2 drinks/day for men, while heavy drinking was ≥ 1 drink/day for women and ≥ 2 drinks/day for men. Physical activity level (< 150, 150–1800, > 1800 MET*min/week) was classified by the total MET-min per week, calculated by multiplying the standard metabolic equivalent (MET) value of each activity by its weekly minutes and then summing across activities. Dietary quality was assessed through 24-hour dietary recalls and HEI scores aligned with the 2015–2020 Dietary Guidelines for Americans were used for the 1999–2014 survey cycles. HEI scores were categorized as < 25%, 25-75%, > 75%, with higher HEI indicating healthier eating habits. Sleeping status included sleeping time (< 5, 5–9, > 9 h) and sleeping disorders (with/without sleeping disorder), obtained from the Sleep-Disorders questionnaire since NHANES 2005–2006. Additionally, depression status (with/without depression) was estimated as an indicator of the mental condition, defined using the nine-item depression screening instrument called the Patient Health Questionnaire (PHQ-9) [19], with a score ≥ 10 considered indicative of depression.

### Demographic and physical variables

Demographic variables included age, gender (male/female), race/ethnicity (Mexican American, Other Hispanic, non-Hispanic White, non-Hispanic Black, other), family economic status (poverty-to-income ratio, ≤ 130%, 130–350%, > 350%), educational level (less than high school, high school or equivalent, college and more), health insurance status (uninsured, any health insurance), and marital status (married, unmarried, other status).

Body mass index (BMI) was calculated as the measured weight in kilograms divided by height in meters squared and categorized as normal (18.5–24.9), overweight (25.0–29.9), obese class I (30.0–34.9), obese class II (35.0–39.9), or obese class III (≥ 40.0). HbA1c level below 7% were considered well-controlled. Uncontrolled hypertension was defined as elevated blood pressure (≥ 140/90 mmHg) measured by a board-eligible physician after the participant had been in a seated position for 5 min of quiet rest. Dyslipidemia was determined by non-high-density lipoprotein (non-HDL) cholesterol, calculated as total measured cholesterol minus HDL cholesterol, with a non-HDL level < 130 mg/dL considered indicative of well-controlled plasma lipids [20].

Diabetic nephropathy (DN) was defined by either a urine albumin to creatinine ratio of 30 mg/g or higher, or estimated glomerular filtration rate less than 60 mL/min/1.73 m^2^. Cardiovascular diseases (CVD) were defined by self-reported any of the following diseases: self-reported congestive heart failure, coronary heart disease, heart attack, or stroke [21].

### Statistical analysis

Data from the NHANES 1999–2000 to NHANES 2017–2018 cycles were analyzed. The distribution of demographic, clinical, and lifestyle characteristics among participants was examined. Lifestyle-related and mental factors were compared by using the student’s *t* test in a weighted linear regression model (for continuous variables) or the weighted Rao-Scott χ^2^ test (for categorical variables) between the two groups (DR and no DR). Given the well-established association between the duration of diabetes and the risk of DR, subgroup analyses were conducted, stratified by diabetic duration to assess differences and variations in lifestyle. A weighted univariable and multivariable logistic regression model was employed to explore the impact of lifestyle on DR, adjusting for potential confounders including gender, age, race/ethnicity, diabetic duration, DN, CVD, HbA1c, blood pressure, lipids control. Appropriate sample weights for the interview sample, examination sample and fasting subsample were utilized. If the level of missing data for primary analyses was less than 10%, a complete case analysis was applied. All statistical analyses were performed using Statistical Analysis Software procedures (SAS version 9.4). A two-sided *p*-value < 0.05 was considered statistically significant.

## Results

### Descriptive statistics

Of the 3482 patients included in the study, 1736 (51.2%) were male and 1746 (48.8%) were female (data was shown as unweighted number and weighted percent). The median age was 52.2 years (IQR: 44.7 to 58.4 years). Demographic and clinical characteristics were presented in Table 1. Participants with self-reported DR exhibited a longer diabetic duration (10.3 years vs. 5.1 years, *p* < 0.001), lower family income (family poverty-to-income ratio ≤ 1.3: 35.3% vs. 25.9%, *p* < 0.001) and lower educational level (less than high school: 27.1% vs. 22.7%, *p* = 0.013), worse HbA1c (< 7%: 53.3% vs. 38.3%, *p* < 0.001), blood pressure (< 140/90 mmHg: 79.1% vs. 71.5%, *p* = 0.004) control and higher prevalence of DN (26.9% vs. 41.4%, *p* < 0.001) and CVD (15.6% vs. 23.4%, *p* < 0.001).

**Table 1:** Characteristics of U.S. 18 to 64-year-old adults with diagnosed diabetes, NHANES 1999–2018*

Characteristic	Overall	no DR	DR	*P* value^†^
Prevalence	3482 (100%)	2678 (79.2)	767 (20.8)	< 0.001
Age, years	52.2 (44.7,58.4)	52.3 (44.7,58.6)	52.0 (44.9,57.6)	0.48
Gender				0.82
Male	1736 (51.2)	1313 (51.1)	407 (51.7)	
Female	1746 (48.8)	1365 (48.9)	360 (48.3)	
Race or ethnicity				0.70
Mexican American	812 (11.0)	617 (11.1)	183 (10.6)	
Other Hispanic	346 (7.0)	269 (7.0)	76 (7.2)	
Non-Hispanic White	959 (55.1)	750 (55.7)	202 (53.2)	
Non-Hispanic Black	1020 (17.9)	785 (17.5)	221 (18.8)	
Others	345 (9.0)	257 (8.7)	85 (10.2)	
Diabetic duration, years	6.0 (2.1,12.5)	5.1 (1.8,10.9)	10.3 (4.3,19.5)	< 0.001
Duration categories				< 0.001
0–10 years	2056 (62.0)	1715 (67.1)	323 (43.3)	
11–19 years	928 (24.6)	668 (23.2)	249 (29.9)	
≥ 20 years	484 (13.4)	283 (9.8)	194 (26.7)	
Educational level				0.013
Less than high school	1200 (23.7)	893 (22.7)	287 (27.1)	
High school or equivalent	770 (24.4)	583 (23.6)	177 (27.2)	
College and more	1508 (51.9)	1200 (53.7)	301 (45.7)	
Poverty-to-income ratio				< 0.001
≤ 1.30	1193 (28.0)	859 (25.9)	320 (35.3)	
1.30–3.50	1159 (35.6)	909 (35.8)	238 (34.8)	
> 3.50	785 (36.4)	642 (38.3)	138 (29.8)	
Health insurance status				0.98
Uninsured	715 (15.6)	558 (15.5)	148 (15.5)	
Any insurance	2754 (84.4)	2109 (84.5)	617 (84.5)	
Marital status				0.15
Married	1927 (59.1)	1492 (59.4)	418 (59.0)	
Unmarried	452 (12.9)	364 (13.4)	79 (10.5)	
Other status	1055 (28.0)	790 (27.2)	256 (30.5)	
BMI categories				0.40
Normal weight (18.5–24.9)	384 (11.1)	278 (10.7)	98 (12.5)	
Overweight (25.0-29.9)	872 (23.4)	674 (22.6)	190 (26.1)	
Class I obesity (30.0-34.9)	867 (26.6)	677 (26.9)	182 (26.0)	
Class II obesity (35.0-39.9)	553 (18.0)	449 (18.6)	99 (15.8)	
Class III obesity (≥ 40)	611 (20.9)	466 (21.2)	138 (19.5)	
Blood pressure				
SBP, mmHg	124.2 (114.5,136.1)	123.9 (114.3,135.8)	125.8 (115.0,138.4)	0.034
DBP, mmHg	73.2 (66.7,79.6)	73.2 (65.8,79.5)	73.2 (65.1,80.0)	0.81
Blood pressure < 140/90 mmHg	2366 (77.5)	1889 (79.1)	477 (71.5)	0.004
Lipid				
Non-HDL, mg/dL	138.8 (111.4,171.1)	138.6 (112.3,169.9)	139.8 (109.4,174.5)	0.95
Non-HDL < 130 mg/dL	1286 (38.6)	1001 (41.1)	285 (42.6)	0.62
Glycemia				
HbA1c, %	7.0 (6.1,8.4)	6.9 (6.1,8.2)	7.5 (6.5,9.2)	< 0.001
HbA1c < 7%	1494 (50.2)	1239 (53.3)	255 (38.3)	< 0.001
Diabetic complications and comorbidity				
DN, %	1119 (29.9)	772 (26.9)	347 (41.4)	< 0.001
CVD, %	633 (17.3)	435 (15.6)	198 (23.4)	< 0.001
Smoking status				0.98
Never smoker	1773 (50.3)	1369 (50.2)	387 (50.6)	
Former smoker	916 (27.2)	694 (27.2)	216 (27.3)	
Current smoker	774 (22.5)	605 (22.6)	163 (22.1)	
Average drinking, median (IQR), unit	0.03 (0.002,0.28)	0.033 (0.004,0.274)	0.013 (0,0.196)	0.39
Drinking status				< 0.001
No drinking	508 (18.1)	380 (16.0)	123 (27.5)	
Mild to moderate drinking	1666 (75.6)	1346 (78.1)	311 (65.0)	
Heavy drinking	158 (6.3)	123 (6.0)	33 (7.5)	
HEI score, median (IQR)	52.2 (51.3,53.1)	51.3 (42.7,60.9)	52.5 (42.9,62.8)	0.38
HEI score				0.24
< 25%	715 (26.5)	535 (26.5)	167 (25.9)	
25-75%	1430 (49.4)	1128 (50.4)	293 (45.8)	
> 75%	714 (24.1)	551 (23.1)	157 (28.3)	
Physical activity status				0.038
< 150 MET-min /week	247 (11.3)	172 (10.0)	69 (16.0)	
150–1800 MET-min /week	1151 (51.1)	920 (52.5)	218 (45.3)	
> 1800 MET-min /week	844 (37.6)	673 (37.5)	166 (38.7)	
Sleeping duration, median (IQR), hours	6.7 (5.7,7.7)	6.7 (5.6,7.8)	6.7 (5.7,7.7)	
Sleeping time duration				< 0.001
< 5 h	211 (6.7)	134 (5.3)	77 (12.6)	
5–9 h	2347 (87.8)	1881 (89.6)	450 (80.4)	
> 9 h	178 (5.5)	129 (5.1)	45 (7.0)	
Sleeping disorder status				0.33
No sleeping disorders	1665 (58.1)	1329 (58.7)	326 (55.8)	
With sleeping disorders	1085 (41.9)	825 (41.3)	251 (44.2)	
Depression status				0.011
No depression	2027 (85.4)	1622 (86.5)	395 (81.4)	
With depression	409 (14.6)	294 (13.5)	110 (18.6)	

^*^Data were shown as unweighted number (weighted percent, %) or weighted median (interquartile range, IQR)

BMI, body mass index. SBP, systolic blood pressure. DBP, diastolic blood pressure. Non-HDL, non-high density lipoprotein cholesterol. HbA1c, glycosylated hemoglobin A1c. DN, diabetic nephropathy. CVD, cardiovascular diseases. HEI, healthy eating index. MET, metabolic equivalent

For interview weights, 3482 patients were included into analysis, data missing are as follows: DR, 37 patients; poverty-to-income ratio, 13 patients; marital status, 48 patients; educational level, 4 patients; diabetic duration, 14 patients; smoking status, 19 patients; CVD data, 25 patients; physical activity status,1240 patients

For mobile examination center weights, 3385 patients were included into analysis, data missing are as follows: BMI, 98 patients; SBP, 154 patients; DBP, 168 patients; HbA1c, 151 patients; DN, 39 patients; non-HDL, 207 patients; drinking status,1053 patients

Sleeping duration, sleeping disorder and depression status was obtained since NHANES 2005–2006. For interview weights, 2754 participants were included into analysis, data missing are as follows: sleeping time duration, 18 patients; sleeping disorder status, 4 patients. For mobile examination center weights, 2683 participants were included into analysis, data missing are as follows:depression status, 247 patients. Day 2 diet recall weight was applied to calculate HEI score and 2859 participants were included into analysis

Statistical significance was defined as *P* < 0.05

^†^*P* value was calculated by Rao-Scott χ^2^ test and student’s *t* test

### Lifestyle and mental health factors in adults aged 18–64 years

Lifestyle and mental health factors were examined including smoking, drinking, diet, physical activity, sleeping, and depression among participants with diabetes, both overall and stratified by self-reported DR status. In the general cohort, 22.5% of patients were current smokers and only 18.1% were non-drinkers. The median HEI score was 52.2 (IQR: 51.3–53.1). 37.6% of participants engaged in higher levels of physical activity (> 1800 MET-min/week) and the median sleeping time duration was 6.7 h. The prevalence of sleeping disorders and depression was 41.9% and 14.6%, respectively.

Considering the established link between diabetes duration and the risk of DR, we conducted a subgroup analysis of lifestyle and psychological status among 18-64-year-old diabetic patients, stratified by disease duration (see Table S1). Notably, with the extension of diabetic duration, the only significant change observed was in alcohol consumption patterns. The proportion of patients who did not drink increased from 16.9 to 20.8%, while excessive drinking decreased from 7.7 to 3.7%. However, no significant improvement were noted in smoking habits, diet quality, sleep status and exercise level, and, notably, the proportion of comorbid depression increased from 13.2 to 20.2% (*p* for trend < 0.001).

### Association between lifestyle and mental health factors with DR

In comparison with patients without DR, self-reported patients exhibited a higher prevalence of heavy drinking habits (7.5% vs. 6.0%, *p* < 0.001), inadequate sleep duration (< 5 h: 12.6% vs. 5.3%, *p* < 0.001), lack of physical activity (< 150 MET-min /week: 16.0% vs. 10.0%, *p* = 0.038), and depression (with depression: 18.6% vs. 13.5%, *p* = 0.011) (see Table 1).

We further conducted a thorough exploration of the impact of lifestyle factors on diabetic retinopathy using univariable and multivariate logistic regression (Table 2). In the univariable logistics regression analysis, mild to moderate alcohol consumption (OR = 0.48, 95%CI: 0.33–0.71, *p* < 0.001) and adequate physical activity (OR = 0.54, 95%CI: 0.32–0.92, *p* = 0.023) were observed to be associated with a reduced risk of DR. In contrast, a sleeping duration of less than 5 h (OR = 2.64, 95%CI: 1.82–3.84, *p* < 0.001) and depression (OR = 1.47, 95%CI: 1.09–1.98, *p* = 0.012) increased the likelihood of developing DR for adults with diabetes. After adjusting for potential confounders including age, sex, race/ethnicity, diabetic duration, HbA1c, blood pressure, lipid control, DN and CVD, mild to moderate drinking (OR = 0.49, 95%CI: 0.32–0.75, *p* = 0.001) and greater physical activity (OR = 0.64, 95%CI: 0.35–0.92, *p* = 0.044) were associated with a lower risk of DR. Conversely, a sleeping duration of < 5 h (OR = 3.18, 95%CI: 2.04–4.95, *p* < 0.001) and the presence of depression (OR = 1.35, 95%CI: 1.06–1.64, *p* = 0.025) were found to elevate the chances of developing DR.

**Table 2:** The association between lifestyle and mental factors and diabetic retinopathy in U.S. 18 to 64-year-old adults with diagnosed diabetes, NHANES 1999–2018*

	Univariable	*P* value^†^	Multivariable	*P* value^†^
Smoking status				
Never smoker (ref)	1		1	
Former smoker	0.996 (0.738,1.346)	0.98	0.924 (0.675,1.264)	0.62
Current smoker	0.972 (0.744,1.271)	0.84	0.912 (0.672,1.237)	0.55
Drinking status				
No drinking (ref)	1		1	
Mild to moderate drinking	0.483(0.331,0.705)	< 0.001	0.487 (0.316,0.748)	0.001
Heavy drinking	0.734 (0.404,1.331)	0.31	0.820 (0.422,1.593)	0.56
HEI score categories				
<25% (ref)	1		1	
25-75%	0.933 (0.648,1.342)	0.71	0.898 (0.612,1.319)	0.58
>75%	1.260 (0.846,1.878)	0.25	1.355 (0.859,2.139)	0.19
Physical activity status				
<150 MET*min/week (ref)	1		1	
150–1800 MET*min/week	0.540 (0.316,0.920)	0.023	0.635 (0.354,0.916)	0.044
>1800 MET*min/week	0.646 (0.393,1.063)	0.085	0.751 (0.433,1.304)	0.31
Sleeping time duration				
< 5 h	2.643 (1.820,3.838)	< 0.001	3.179 (2.042,4.950)	< 0.001
5–9 h (ref)	1		1	
> 9 h	1.534 (0.808,2.910)	0.19	1.628 (0.836,3.169)	0.15
Sleeping disorder status				
No sleeping disorders (ref)	1		1	
With sleeping disorder	1.128 (0.882,1.442)	0.33	1.208 (0.898,1.624)	0.21
Depression status				
No depression (ref)	1		1	
With depression	1.472 (1.092,1.983)	0.012	1.349 (1.059,1.639)	0.025

* Data was shown as OR (95% CI). Ref, reference; OR, odds ratio; CI, confidence interval; MET, metabolic equivalent; HEI, healthy eating index. Odds ratios were adjusted for age, sex, race/ethnicity, diabetic duration, diabetic nephropathy, cardiovascular diseases, HbA1c control, blood pressure control and non-high density lipoprotein cholesterol control. Statistical significance was defined as P < 0.05

†*P* value was calculated by weighted logistic regression

## Discussion

In this large population-based study, we provided an initial description of the prevalence of diabetic retinopathy and lifestyle-related factors among 18 to 64-year-old adults with self-reported diagnosed diabetes. Moreover, we explored the impact of lifestyle and mental factors on DR. The findings highlighted that the individuals with DR tend to exhibit poorer lifestyle management and a more precarious mental condition. Specifically, mild to moderate alcohol consumption and greater physical activity were protective factors against DR, whereas sleep deprivation and depression were associated with an increased risk.

From our study, the overall prevalence of DR among working-age adults was found to be 20.8%, which is lower than the global prevalence reported in 2012 [22]. Several reasons might account for this difference. First, our study specifically included adults under 65 years old, suggesting a likely shorter diabetes duration than in the general patient population, potentially resulting in less progression of DR pathophysiology. Second, as a nationally cross-sectional research endeavor, data from NHANES may be limited in accurately representing global prevalence trends. Additionally, the prevalence estimates could be due to the reliance on self-reported diabetes instead of incorporating self-report and HbA1c, potentially resulting in an underestimation [23–26].

Demographic and socioeconomic factors have been shown to significantly influence diabetic complications, potentially mediated through lifestyle factors influencing healthcare tendentiousness and self-management engagement [27]. In our study, patients diagnosed with DR exhibited a worse socioeconomic status, including lower education and family income level, contributing to spatial heterogeneity and uneven health care. Although diabetic duration is a well-recognized risk factor for DR [23], our observation revealed that [28], only alcohol consumption significantly reduced over time. Few studies have explored the impact of diabetic duration on lifestyle. Longer diabetic duration was associated with poorer glycemic control, while early intervention could promote positive outcomes in pre-diabetes [29]. However, our data suggested that increased duration didn’t improve the patient’s self-management awareness or healthy lifestyle preferences, but rather increased the prevalence of depression. Therefore, it is critically important to deliver more robust health education to patients with long-term disease.

Numerous studies have consistently demonstrated that adopting healthy lifestyles can reduce the incidence of diabetes and its associated complications. In alignment with previous research, our research indicated that smoking tends to be a risk factor of DR. This conclusion Is supported by earlier investigations, including a meta-analysis involving 73 studies, which found a significant association between smoking and increased risk of DR in type 1 diabetes [30]. Notably, although smoking cessation le to a reduction of prevalence of DR, individuals who quit smoking still exhibited a higher prevalence of DR and CVD compared to non-smokers [31]. In our study, we observed that mild to moderate drinking appears to confer protection against diabetic retinopathy. Similar findings have been reported in prior studies. The EURODIAB Prospective Complications Study, which followed 1857 patients with diabetes, established a connection between moderate alcohol consumption and a decreased risk of proliferative retinopathy, neuropathy and macroalbuminuria. Furthermore, moderate alcohol consumption, defined as one to three drinks a day, has been associated with reduced cardiovascular disease risk in the general population [32, 33]. However, it’s important to note that some publications failed to identify a significant association between alcohol consumption and diabetic complications [34].

From our study, a higher HEI score reflecting better dietary quality did not show an association with a lower DR risk. However, emerging evidence suggests that improved diet quality is associated with other diabetes complications including DN and coronary heart disease. For instance, among 461 youth and young adults with type 1 diabetes, a borderline inverse association was observed between the HEI score and microalbuminuria (OR = 0.83, *p* = 0.07) [35]. Additionally, a cross-sectional study of 358 type 2 diabetic adults found that higher HEI scores were associated with a lower 10-year CVD risk through improved glucose/lipid control [36].

In contrast, our study indicated that greater physical activity is associated with reduced risk of DR, which was in line with previous studies. The inverse association between physical activity and the risk of diabetic complications may be attributed to reduced inflammation and elevated levels of 25-OH-Vitamin D3 [37].In this study, sleeping for less than 5 h was associated with an increased risk of DR. Sleep duration demonstrated a U-shaped association with diabetic vascular complications, with 6–8 h recommended for the lowest risk [38]. Beyond sleep deprivation, poor sleep quality has also been shown to impact the incidence of diabetic complication [39]. Potential mechanisms underlying these associations may involve sleep deprivation decreasing insulin sensitivity and enhancing systemic inflammatory responses due to circadian rhythm disruption [40].

Our observation establishes a link between depression and an increased risk of DR. This aligns with the findings reported in previous study where a meta-analysis of 27 studies revealed a significant correlation between depression and diabetes complications [41]. Depression influences diabetes control through biological and behavioral pathways, hypothalamic-pituitary-adrenal axis activation, sympathetic stimulation, inflammation, and subsequent poor glycemic control [42]. Additionally, depression can impair glycemic control by negatively affecting self-care practices, medication adherence, and preventive care. Notably, young patients with diabetes are more likely to experience depression [43], highlighting the need to address their psychological health as part of a comprehensive diabetes management approach.

Our study aimed to estimate the prevalence of DR and examine the impact of lifestyle and mental health factors among U.S. participants aged 18 to 64 years. However, there are several limitations that may hinder the generalizability of our findings. First, the reliance on self-reported diabetic retinopathy and lifestyle factors may introduce recall bias, as a proportion of individuals with DR may be unaware of their diagnosis [44]. A study by Kristen et al. reported that 10.6% of participants older than 40 years who underwent fundus photography in NHANES were unaware of their DR diagnosis, including 23.1% with self-reported diabetes and 6.8% reported without a diabetes diagnosis. Besides, the study is cross-sectional in nature, and further cohort studies are needed to confirm our findings. Nevertheless, it is important to note that this study included a large representative sample population selected through a complex, multistage sampling method. The use of objective statistical methods and adjustment for various interactions aimed to mitigate potential bias, thereby enhancing the reliability of the association between lifestyle factors and DR.

## Conclusions

Collectively, adults aged 18–64 years with DR demonstrated a higher prevalence of lifestyle risk factors, including excessive drinking, sleep disorders, unhealthy diet, and a more precarious mental status including the presence of depression. Beyond the management of metabolic risk factors, there is a clear need to promote healthy lifestyles within this population to effectively prevent the onset and progression of diabetic retinopathy.

### Electronic supplementary material

Below is the link to the electronic supplementary material.

Supplementary Material 1

## Data Availability

The code and data generated during the study were available from the corresponding author on reasonable request. And the original data was public on the NHANES website (https://www.cdc.gov/nchs/nhanes/index.htm).

## Abbreviations

DR: Diabetic Retinopathy
NHANES: National Health and Nutrition Examination Survey
HbA1c: Glycosylated hemoglobin A1c
SBP: Systolic Blood Pressure
DBP: Diastolic Blood Pressure
Non-HDL: Non-high-Density Lipoprotein
DN: Diabetic Nephropathy
HEI: Healthy Eating Index
MET: Metabolic Equivalent
PHQ: Patient Health Questionnaire
CVD: Cardiovascular Diseases
BMI: Body Mass Index
OR: Odds Ratio
CI: Confidential Interval
IQR: Inter-quartile Range
