# Supplementary material for: Modifiable lifestyle, mental health status and diabetic retinopathy in U.S. adults aged 18–64 years with diabetes: a population-based cross-sectional study from NHANES 1999–2018
Source: BMC Public Health. 2024 Jan 2;24:11. doi: 10.1186/s12889-023-17512-8 (PMC10759477; doi:10.1186/s12889-023-17512-8)
Supplement: Supplementary file 1 — Supplementary Material 1 [file 12889_2023_17512_MOESM1_ESM.docx]

**Modifiable lifestyle, mental health status and diabetic retinopathy in adults aged 18-64 years with diabetes: A population-based cross-sectional study from NHANES 1999-2018**

Bo Li, Chuandi Zhou, Chufeng Gu, Xiaoyun Cheng, Yujie Wang, Chenxin Li, Mingming Ma, Ying Fan, Xun Xu, Haibing Chen, Zhi Zheng

Corresponding author: Zhi Zheng, Department of Ophthalmology, Shanghai General Hospital, Shanghai Jiao Tong University School of Medicine, Email address: zzheng88@sjtu.edu.cn.

Table S1. Diabetic duration-stratified lifestyles characteristics of U.S. 18 to 64-year-old adults with diagnosed diabetes, NHANES 1999-2018

Table S2. The association between lifestyle factors and diabetic retinopathy in U.S. 18 to 64-year-old adults with diagnosed diabetes by diabetic duration, NHANES 1999-2018

| Table S1. Diabetic duration-stratified lifestyles characteristics of U.S. 18 to 64-year-old adults with diagnosed diabetes, NHANES 1999-2018^*^ | | | | | |
| --- | --- | --- | --- | --- | --- |
| Characteristic | 0-10 years | 11-19 years | ≥20 years | P value^†^ | P for trend |
| Smoking status |  |  |  | 0.50 | 0.69 |
| Never smoker | 1045(50.5) | 480(51.0) | 243(48.5) |  |  |
| Former smoker | 518(26.0) | 252(28.6) | 141(30.5) |  |  |
| Current smoker | 478(23.6) | 192(20.4) | 100(20.9) |  |  |
| Drinking status |  |  |  | 0.023 | 0.012 |
| No drinking | 294(16.9) | 134(19.7) | 76(20.8) |  |  |
| Mild to moderate drinking | 1007(75.5) | 447(76.3) | 210(75.5) |  |  |
| Heavy drinking | 109(7.7) | 31(3.9) | 18(3.7) |  |  |
| HEI score, mean (95%CI) | 51.8(50.4,53.0) | 53.2(51.7,54.7) | 52.7(50.9,54.6) | 0.20 | 0.20 |
| HEI score |  |  |  | 0.52 | 0.25 |
| <25% | 442(27.7) | 178(26.3) | 94(21.7) |  |  |
| 25%-75% | 827(49.0) | 382(47.4) | 213(54.1) |  |  |
| >75% | 431(23.4) | 196(26.3) | 87(24.1) |  |  |
| Physical activity status |  |  |  | 0.49 | 0.17 |
| <150 MET-min /week | 159(11.8) | 54(10.1) | 33(11.0) |  |  |
| 150-1800 MET-min /week | 700(52.4) | 301(48.9) | 146(48.2) |  |  |
| >1800 MET-min /week | 493(35.7) | 242(41.0) | 108(40.8) |  |  |
| Sleeping duration, median (IQR), hours | 6.7(5.7,7.7) | 6.6(5.6,7.7) | 6.9(5.8,7.8) | 0.29 | 0.29 |
| Sleeping time duration |  |  |  | 0.55 | 0.59 |
| < 5 hours | 115(6.3) | 59(7.7) | 34(5.6) |  |  |
| 5-9 hours | 1372(88.7) | 669(86.0) | 298(88.2) |  |  |
| >9 hours | 92(5.0) | 57(6.2) | 28(6.2) |  |  |
| Sleeping disorder status |  |  |  | 0.093 | 0.64 |
| No sleeping disorders | 988(59.6) | 461(53.1) | 209(61.5) |  |  |
| With sleeping disorders | 601(40.4) | 326(46.9) | 153(38.5) |  |  |
| Depression status |  |  |  | 0.14 | 0.05 |
| No depression | 1200(86.8) | 575(84.8) | 244(79.8) |  |  |
| With depression | 218(13.2) | 124(15.2) | 67(20.2) |  |  |
| ^*^ Data were shown as unweighted number (weighted percent, %) or median (interquartile range, IQR). HEI indicates healthy eating index. MET indicates metabolic equivalent. Statistical significance was defined as P < 0.05.  ^†^ P value was calculated by Rao-Scott Chi-square test or student’s t test in weighted linear regression. | | | | | |

| Table S2. The association between lifestyle factors and diabetic retinopathy in U.S. 18 to 64-year-old adults with diagnosed diabetes by diabetic duration, NHANES 1999-2018^*^ | | | | | | |
| --- | --- | --- | --- | --- | --- | --- |
|  | 0-10 years | P value | 11-19 years | P value | ≥20 years | P value^†^ |
| Smoking status |  |  |  |  |  |  |
| Never smoker (ref) | 1 |  | 1 |  | 1 |  |
| Former smoker | 0.665(0.422,1.048) | 0.078 | 1.266(0.713,2.247) | 0.42 | 1.027(0.517,2.039) | 0.94 |
| Current smoker | 0.898(0.587,1.373) | 0.62 | 1.116(0.663,1.878) | 0.68 | 0.652(0.296,1.438) | 0.29 |
| Drinking status |  |  |  |  |  |  |
| No drinking (ref) | 1 |  | 1 |  | 1 |  |
| Mild to moderate drinking | 0.394(0.224,0.695) | 0.002 | 0.444(0.218,0.905) | 0.026 | 0.841(0.362,1.954) | 0.69 |
| Heavy drinking | 0.898(0.395,2.040) | 0.80 | 0.256(0.076,0.896) | 0.029 | 0.778(0.178,3.398) | 0.74 |
| HEI score categories |  |  |  |  |  |  |
| <25% | 1 |  | 1 |  | 1 |  |
| 25%-75% | 0.926(0.5545,1.701) | 0.89 | 0.624(0.333,1.168) | 0.14 | 1.173(0.490,2.808) | 0.72 |
| >75% | 1.091(0.547,2.173) | 0.80 | 1.331(0.664,2.669) | 0.42 | 2.752(0.990,7.653) | 0.05 |
| Physical activity status |  |  |  |  |  |  |
| <150 MET*min/week (ref) | 1 |  | 1 |  | 1 |  |
| 150-1800 MET*min/week | 0.491(0.261,0.922) | 0.027 | 0.705(0.264,1.884) | 0.48 | 1.040(0.320,3.383) | 0.95 |
| >1800 MET*min/week | 0.612(0.335,1.119) | 0.11 | 1.341(0.526,3.416) | 0.54 | 0.376(0.108,1.303) | 0.12 |
| Sleeping time duration |  |  |  |  |  |  |
| < 5 hours | 2.672(1.479,4.827) | 0.001 | 4.928(2.168,11.201) | <0.001 | 3.344(0.876,12.770) | 0.077 |
| 5-9 hours (ref) | 1 |  | 1 |  |  |  |
| >9 hours | 1.712(0.594,4.933) | 0.32 | 1.374(0.609,3.101) | 0.44 | 3.116(0.690,14.067) | 0.14 |
| Sleeping disorder status |  |  |  |  |  |  |
| No sleeping disorders (ref) | 1 |  | 1 |  | 1 |  |
| With sleeping disorder | 1.210(0.812,1.804) | 0.34 | 1.046(0.652,1.678) | 0.85 | 1.505(0.686,3.300) | 0.30 |
| Depression status |  |  |  |  |  |  |
| No depression (ref) | 1 |  | 1 |  | 1 |  |
| With depression | 1.396(1.007,1.785) | 0.022 | 1.973(1.177,3.305) | 0.01 | 0.586(0.252,1.359) | 0.21 |
| ^*^ Data was shown as OR (95% CI). Ref, reference; OR, odds ratio; CI, confidence interval; MET, metabolic equivalent; HEI, healthy eating index. Odds ratios were adjusted for age, sex, race/ethnicity, diabetic duration, diabetic nephropathy, cardiovascular diseases, HbA1c control, blood pressure control and non-high density lipoprotein cholesterol control. Statistical significance was defined as P < 0.05.  ^†^ P value was calculated by weighted logistic regression. | | | | | | |
